# Bidirectional association between depression and cognition in Chinese middle-aged and older women: a 10-year longitudinal study

**DOI:** 10.3389/fpsyt.2025.1531202

**Published:** 2025-05-02

**Authors:** Xiaoxu Jiang, Zheng Jiang

**Affiliations:** ^1^ Maternal and Child Health Development Research Center, Shandong Provincial Maternal and Child Health Care Hospital Affiliated to Qingdao University, Jinan, China; ^2^ Department of Neurosurgery, Qilu Hospital, Cheeloo College of Medicine and Institute of Brain and Brain-Inspired Science, Shandong University, Jinan, China; ^3^ Shandong Key Laboratory of Brain Health and Function Remodeling, Jinan, China

**Keywords:** depression, cognition, Chinese middle-aged and older females, CHARLS, longitudinal

## Abstract

**Background:**

Women’s health is an important issue worldwide, and as the population is aging, the health of middle-aged and older women is becoming increasingly vital. Although many studies have examined the relationship between cognition and depression, few studies have been conducted specifically with middle-aged and older women. This study used a longitudinal approach to examine the bidirectional relationship between cognition and depression in middle-aged and older women.

**Methods:**

The study used three waves (2011, 2015, and 2020) of data from the China Health and Retirement Longitudinal Study (CHARLS), enrolling a total of 4,618 middle-aged and older women aged over 45 years. Participants’ general demographic characteristics were analyzed descriptively, and changes in study variables were measured using repeated-measures analysis of variance. Generalized estimating equation (GEE) and cross-lagged models were used to investigate the longitudinal relationship between depression and cognition.

**Results:**

The results of the GEE and cross-lagged models revealed that previous cognitive problems lead to future depression and prior depressive conditions affect subsequent cognition.

**Conclusions:**

There is a bidirectional relationship between depression and cognition in middle-aged and older women over time. More clinical mechanisms need to be explored in the future.

## 1 Introduction

The process of population aging in China is progressing rapidly, and the situation is becoming increasingly severe (1). According to data from the Seventh National Population Census of China, approximately 300 million women aged over 45 account for 20% of the total population (2). Studies reveal that elderly Chinese women are more likely to experience poorer health outcomes compared to their male counterparts (3, 4). Furthermore, the prevalence of adverse health conditions shows an age-related increase among women. Although women across all age groups face various health challenges, middle-aged and elderly women appear to be particularly vulnerable (5, 6).

Depressive disorder is a psychiatric condition characterized by persistent low mood and/or diminished interest in daily activities. Epidemiological studies demonstrate a 50% higher prevalence rate in women compared to men (7). Notably, Yue’s national survey (8) reveals that 22.3% of Chinese middle-aged and elderly women exhibit clinically significant depressive symptoms, with particularly elevated rates observed in rural populations (25.7%) and specific female subgroups (27.9%). Postmenopausal estrogen depletion has been identified as a significant biological risk factor for depression onset in perimenopausal women (9). In older populations, depression predominantly manifests through interacting pathways including multimorbidity patterns, chronic pain syndromes, social isolation, and adverse life experiences (10, 11).

Cognitive impairment is characterized by neurodegenerative changes affecting memory and executive function (12). Impaired cognitive function is a common complaint among older women seeking care in mental health and medical facilities (13). Epidemiological data from China indicate dementia prevalence rates of approximately 10% in the elderly population, with mild cognitive impairment affecting 20%–30% of this demographic (14, 15). Notably, a longitudinal cohort study in Jamaica identified female gender and advanced age as independent risk factors for dementia progression (16).

Several studies have explored the relationship between depression and cognition. Lyu’s study demonstrated a significant correlation between depression and cognitive impairment in both sexes (17). Cross-sectional analyses showed that women and low cognitive levels were related to higher depression (18). It is even noted that in humans, women are more at risk of experiencing greater cognitive decline in Alzheimer’s disease and depression (19). Estrogen changes in menopausal women may damage neurons that regulate emotions and cognition (20). Epidemiological and clinical evidence indicates that emotion and cognition in older adults are clinically interrelated, and common neurobiological mechanisms may underlie both groups of diseases (21).

In summary, given the importance of depression and cognition in human health development, several studies have explored their relationship. However, existing research has primarily focused on population-wide or maternal populations, with limited attention to middle-aged and older women. Additionally, most studies have adopted a unidirectional approach, rarely exploring bidirectional relationships. To address these gaps, this study employs longitudinal analyses to investigate the bidirectional relationship between depression and cognition among middle-aged and older women.

## 2 Methods

### 2.1 Study population

The data for this study came from the China Health and Retirement Longitudinal Study (CHARLS). CHARLS is a tracking survey that is representative of mainland China’s population aged 45 years and older. Its national baseline survey was conducted in 2011, and four rounds of tracking surveys with routine questionnaires were conducted in 2013, 2015, 2018, and 2020, respectively (22).

The CHARLS baseline survey used a multi-stage (county/district-village/residence-household) PPS (probability proportional to size sampling) random sampling method. In the first stage, all counties and districts in the country, except Tibet, were ranked by urban–rural attributes and GDP (gross domestic product) per capita within each of the eight regions, and then 150 counties or districts were selected with a probability proportional to population size. In the second stage, within each sampled county, three secondary sampling units (villages or communities) are randomly selected with a probability proportional to population size. Thus, CHARLS is nationally as well as regionally representative. After the sampling process described above, the baseline sample for CHARLS was distributed among 450 villages and neighborhoods in 28 provinces and 150 districts and counties across the country (23).

**Figure 1:**
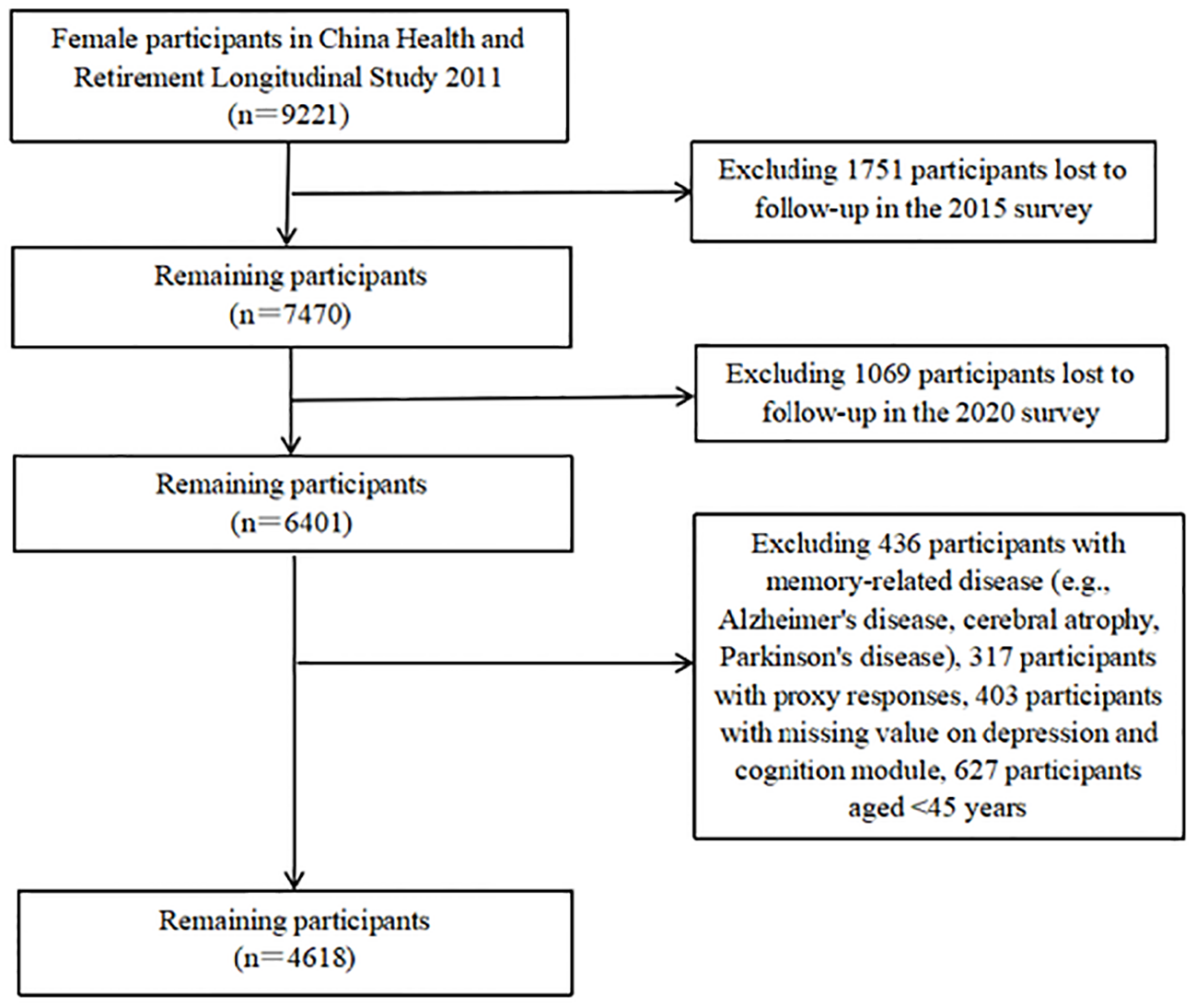
Sample selection process in this study.

This study used the CHARLS baseline (2011), third round (2015), and fifth round (2020) data; participants over 45 were defined as middle-aged and older women. A sample size of 4,618 women were included in this study. The inclusion and exclusion criteria for the sample were as follows: (1) There were 9,221 female participants at baseline, 1,751 participants failed to follow-up in the 2015 survey, and 1,069 participants failed to follow-up in the 2020 survey; (2) a total of 753 participants with memory-related diseases (e.g., Alzheimer’s disease, cerebral atrophy, and Parkinson’s disease) and proxy response were excluded; (3) a total of 403 participants with missing data on depression and cognition modules were removed; (4) a total of 627 participants under 45 years at baseline were likewise excluded. The specific sample selection process is shown in **Figure 1**.

### 2.2 Measurements

In this study, socio-demographic information includes age and education, among others. Depression was measured using the 10-item Centre for Epidemiological Studies, Depression Scale (CES-D-10). Cognition was assessed by orientation and attention, word recall, and visuospatial abilities.

#### 2.2.1 Demographic characteristics

General demographic information included age, marital status (married with spouse present, married but not living with spouse temporarily, separated or divorced, widowed, and never married), education (no formal education, primary or incomplete primary education, secondary school, college, and above), residence (city or town, village), self-assessed health (very good, good, fair, poor, and very poor), and self-assessed memory (excellent, very good, good, fair, and poor).

#### 2.2.2 Depression

The CES-D was developed in 1977 by Radloff at the National Institute of Mental Health in the United States (24). The scale is widely used in epidemiological surveys to screen out subjects with depressive symptoms for further examination and diagnosis. The CES-D-10 is a short version of the scale, which has been validated in Chinese middle-aged and older adults with good reliability and validity (25). There are 10 items in the CES-D-10, with each item ranging from 0 (little) to 3 (most of the time). The total score ranges from 0 to 30, ≥10 suggests a risk of depressive symptoms, and higher scores indicate more severe depressive symptoms (26).

#### 2.2.3 Cognition

Cognition was assessed by orientation and attention, word recall, and visuospatial abilities. First, participants were assessed for orientation and attention by asking for the year (one point), month (one point), day (one point), day of the week (one point), and current season (one point), then asked to subtract 7 (up to five times) from 100 consecutively (five points). Each correct answer scores 1 point, and the total score for these two sections is 10 points. Second, the respondents were able to immediately repeat the 10 Chinese nouns that had just been read to them (immediate recall was 10 points) and recall the same list of words a few minutes later (delayed recall was 10 points). The total mean score for both parts was 10. It is generally accepted that the average score of immediate recall and delayed recall is an episodic memory measure. Finally, visuospatial ability was tested by reproducing two pictures of overlapping pentagons, with participants scoring one point if they were successful in drawing it (one point). Cognitive ability scores ranged from 0 to 21; participants with higher scores were considered to have better cognitive functioning (Yurun Du et al., 2023; Yushan Du et al., 2023; Yang et al., 2020).

### 2.3 Statistical analysis

Descriptive analyses were used to demonstrate demographic characteristics at baseline, and percentages were used for qualitative variables. Repeated-measures analysis of variance (RM-ANOVA) was used to compare depression and cognition in 2011, 2015, and 2020. Longitudinal analyses use generalized estimating equation (GEE), including demographic variables. GEE has a wider application, especially for analyses of longitudinal data. It considers intra-individual correlations, focuses on estimating the average effect at the aggregate level, and does not make hypothetical estimates, making inferences about conclusions more reliable (27). The cross-lagged model is mainly applied to estimate the intertemporal effects of two or more variables at multiple points in time. It includes auto-regression effects and cross-lagged effects (28). To assess the fit of the model, we used the following indicators: (1) χ^2^/degree of freedom (df), (2) root mean square error of approximation (RMSEA), and (3) comparative fit index (CFI) (29). A significance level of 5% was used for all statistical tests. IBM SPSS Statistics, version 26.0 (IBM Corp., Armonk, NY, USA) and AMOS 24 were used to carry out the statistical analyses.

### 2.4 Ethics approval and consent to participate

Ethics approval for the study was granted by the Ethical Review Committee of Peking University. The IRB approval number is IRB00001052-11015. Informed consent was obtained at the time of participation. All methods of this study were performed in accordance with the relevant guidelines and regulations. All experimental protocols were approved by the Institutional Review Board at Peking University.

## 3 Results

This study included a total of 4,618 Chinese middle-aged and elderly women. Baseline characteristics of the participants are summarized in **Table 1**. Participants had a mean age of 57.8 ± 7.76 years. At baseline, 87.6% of participants were married, and 77.5% had a primary school education or below. Most participants lived in villages (70.2%). Approximately half of the participants rated their health as fair (53.2%), while 44.6% reported their memory as fair.

**Table 1:** General demographic characteristics of participants in baseline.

Variables	2011 (*n* = 4,618) *n* (%)
Age, mean ± SD	57.80 ± 7.76
Marital status	
Married with spouse present	3,797 (82.2)
Married but not living with spouse temporarily for reasons such as work	250 (5.4)
Separated or divorced	39 (0.8)
Widowed	526 (11.4)
Never married	6 (0.2)
Educational level	
No formal education	1,866 (40.4)
Primary or incomplete primary education	1,713 (37.1)
Secondary school	962 (20.8)
College and above	77 (1.7)
Residence	
City or town	1,374 (29.8)
Village	3,244 (70.2)
Self-assessed health	
Very good	257 (5.6)
Good	740 (16.0)
Fair	2,457 (53.2)
Poor	1,001 (21.7)
Very poor	163 (3.5)
Self-assessed memory	
Excellent	11 (0.2)
Very good	195 (4.2)
Good	519 (11.2)
Fair	2,053 (44.6)
Poor	1,840 (39.8)

**Table 2** demonstrates the changes in depression and cognition. The mean for depression in 2011 was 9.18 (SD = 6.50), that for 2015 was 9.16 (SD = 6.63), and that for 2020 was 10.17 (SD = 6.71). The mean for cognition in 2011 was 9.60 (SD = 4.30), that for 2015 was 9.21 (SD = 4.49), and that for 2020 was 9.88 (SD = 4.94). Groups were significantly different based on *post-hoc* tests. The changing profile of depression and cognition remained relatively stable.

**Table 2:** Depression and cognition in 2011, 2015, and 2020.

Variables	Depression (CES-D)	Cognition
Repeated-measures analysis of variance (RM-ANOVA)		
2011 (mean ± SD)	9.18 ± 6.50	9.60 ± 4.30
2015 (mean ± SD)	9.16 ± 6.63	9.21 ± 4.49
2020 (mean ± SD)	10.17 ± 6.71	9.88 ± 4.94
F	64.817	73.797
*P*	<0.001	<0.001
*Post-hoc* analysis (Depression)		
2011–2015 (*p*)	0.856	
2011–2020 (*p*)	<0.001	
2015–2020 (*p*)	<0.001	
*Post-hoc* analysis (Cognition)		
2011–2015 (*p*)	<0.001	
2011–2020 (*p*)	<0.001	
2015–2020 (*p*)	<0.001	

**Table 3** illustrates the findings of the GEE analysis, with depression established as the dependent variable. The results demonstrate that age, marital status, education, residence, self-assessed health, self-assessed memory, and cognition are significantly associated with depression. Participants who were middle-aged and widowed, had no formal education, lived in a city or town, and had poorer self-assessed health and memory were more depressed. Cognition and depression were significantly correlated (β = −0.197, *p* < 0.001). over time, participants with poorer cognitive status became more depressed.

**Table 3:** GEE results of cognition and depression (dependent variable: depression).

Variables	β	SE	95% CI	*p*
Age	−0.032	1.284	5.834 to 10.865	0.002
Marital status				
Married with spouse present	1.0			
Married but not living with spouse temporarily for reasons such as work	0.213	0.324	−0.422 to 0.847	0.511
Separated or divorced	1.784	0.928	−0.035 to 3.602	0.055
Widowed	1.038	0.230	0.587 to 1.489	<0.001
Never married	1.905	1.147	−0.342 to 4.152	0.097
Educational level				
No formal education	1.0			
Primary or incomplete primary education	0.276	0.170	−0.057 to 0.610	0.105
Secondary school	−0.202	0.221	−0.635 to 0.231	0.361
College and above	−1.037	0.449	−1.917 to −0.157	0.021
Residence				
City or town	1.0			
Village	0.654	0.158	0.345 to 0.963	<0.001
Self-assessed health				
Very good	1.0			
Good	0.842	0.292	0.270 to 1.415	0.004
Fair	2.365	0.270	1.836 to 2.894	<0.001
Poor	4.905	0.309	4.300 to 5.510	<0.001
Very poor	7.211	0.494	6.243 to 8.180	<0.001
Self-assessed memory				
Excellent	1.0			
Very good	0.082	1.153	−2.178 to 2.342	0.943
Good	0.274	1.139	−**1**.959 to 2.507	0.810
Fair	1.007	1.130	−1.207 to 3.222	0.373
Poor	2.569	1.135	0.345 to 4.794	0.024
Cognition	−0.197	0.017	−0.230 to −0.163	<0.001

**Table 4** presents the results of the GEE with cognition as the dependent variable. Age, marital status, education, residence, self-assessed health, and depression are significantly correlated. Cognitive functioning was worse among participants who were older, widowed, or unmarried, had no formal education, lived in a village, and had poorer self-assessed health. Depression and cognition were significantly correlated (β = −0.068, *p* < 0.001). Over time, more depressed participants had poorer cognitive functioning.

**Table 4:** GEE results of cognition and depression (dependent variable: cognition).

Variables	β	SE	95% CI	*p*
Age	−0.076	0.006	−0.088 to −0.065	<0.001
Marital status				
Married with spouse present	1.0			
Married but not living with spouse temporarily for reasons such as work	−0.349	0.188	−0.717 to 0.019	0.063
Separated or divorced	−0.392	0.389	−1.153 to 0.370	0.313
Widowed	−0.370	0.137	−0.639 to −0.101	0.007
Never married	−1.781	0.741	−3.234 to −0.328	0.016
Educational level				
No formal education	1.0			
Primary or incomplete primary education	3.301	0.101	3.102 to 3.499	<0.001
Secondary school	5.238	0.120	5.003 to 5.473	<0.001
College and above	6.607	0.245	6.126 to 7.088	<0.001
Residence				
City or town	1.0			
Village	−1.081	0.095	−1.267 to −0.895	<0.001
Self-assessed health				
Very good	1.0			
Good	−0.296	0.196	−0.681 to 0.089	0.132
Fair	−0.441	0.178	−0.789 to −0.093	0.013
Poor	−0.430	0.196	−0.814 to −0.047	0.028
Very poor	−0.687	0.299	−1.272 to −0.102	0.021
Self-assessed memory				
Excellent	1.0			
Very good	1.189	1.060	−0.889 to 3.266	0.262
Good	1.139	1.051	−0.922 to 3.199	0.279
Fair	1.103	1.047	−0.949 to 3.156	0.292
Poor	0.010	1.049	−2.046 to 2.066	0.992
Depression	−0.068	0.006	−0.079 to −0.056	<0.001

**Figure 2:**
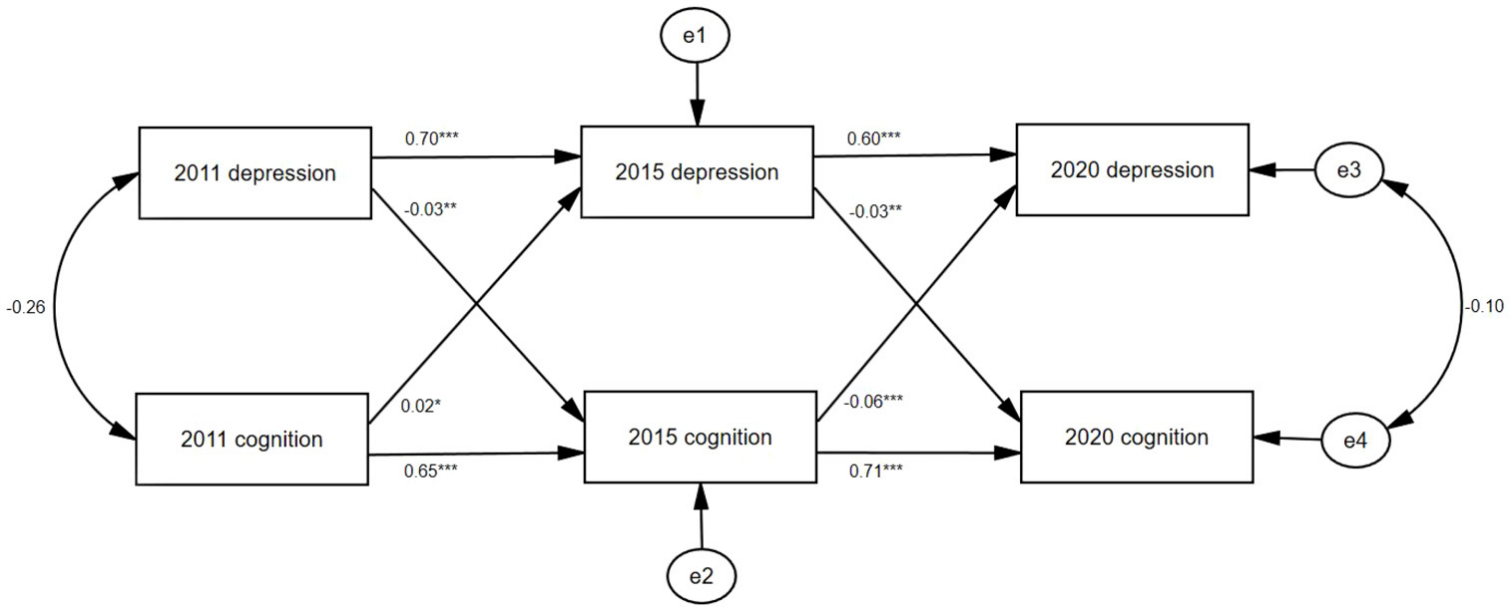
Cross-lagged model of depression and cognition. e1, e2, e3, and e4: potential errors; *p < 0.05, **p < 0.01, ***p < 0.001.

**Figure 2** displays the results of the cross-lagged model. Cross-lagged regression showed that depression in 2011 had a significant effect on cognition in 2015 (β = −0.03, *p* < 0.05) and depression in 2015 had a significant effect on cognition in 2020 (β = −0.03, *p* < 0.05). Cognition in 2011 had a significant effect on depression in 2015 (β = 0.02, *p* < 0.01) and cognition in 2015 had a significant effect on depression in 2020 (β = −0.06, *p* < 0.001). Auto-regression effects in depression were significant (2011 to 2015: β = 0.70, *p* < 0.001; 2015 to 2020: β = 0.60, *p* < 0.001). Auto-regression effects in cognition were significant (2011 to 2015: β = 0.65, *p* < 0.001; 2015 to 2020: β = 0.71, *p* < 0.001). The fit metrics for this model were as follows: χ^2^/df >5, RMSEA = 0.10, and CFI = 0.80; the χ^2^/df is large due to the sample size, but the model is generally acceptable (30).

## 4 Discussion

This study is one of the few that examine the bidirectional relationship between depression and cognition in Chinese middle-aged and older women. The latest round of CHARLS (2020) data was used. Three rounds of longitudinal data were used to demonstrate the bidirectional relationship between depression and cognition. A key finding is the bidirectional relationship between depression and cognition: higher depression severity predicted worse cognitive function, and conversely, lower cognitive function predicted increased depressive symptoms. These findings highlight that we need to improve the health conditions among middle-aged and older women by improving depression and cognitive status.

It was found that women in China in the 1960s had a very low level of education, and education had a significant effect on depression and cognition. This is in line with many studies (31–33). Having an extra year of schooling has a huge and significant protective effect on mental health and memory (34). Mexican research has revealed that women’s education has an even higher impact on cognition, with more pronounced cognitive benefits in the future (35).

The bidirectional relationship between cognition and depression is consistent with the results of longitudinal studies in Europe (36) and Korea (37). In addition, Rebecca’s study found a stable negative correlation between depression and cognition only in women (38). As age increases, it is inevitable to experience deteriorating cognitive functions, especially reduced memory and impaired judgment, which can affect social functions such as interpersonal interactions in work, life, and family, and further lead to depression or other negative mental issues (39). Shaikh’s study similarly confirms that memory changes in older adults could have an impact on daily life, such as limitations on lifestyle activities and negative emotions (40). There are also studies that have come to the opposite conclusion, suggesting that early cognitive impairment does not lead to feelings of depression in later life, perhaps because of differences in study populations and samples (41).

The findings of this study indicate that for middle-aged and older women, previous depression affects later cognition, which is consistent with the findings of many previous studies (42). The explanation in Jutta’s study is that depressive symptoms may trigger hypercortisolism, increase autonomic responses associated with cardiovascular disease, lead to inflammation, and, in turn, lead to neurotoxicity. These processes accelerate cognitive decline in the aging process (43). Yuan’s comparable study in China found that depression preceded cognitive decline in older adults, and initial depression affected subsequent cognitive function, particularly immediate and delayed recall, converging with the results of the present study (44). However, a study based on the UK female birth cohort showed that most aspects of midlife mental health were not prospectively associated with cognition (45).

In conclusion, the data used in this study further support the feasibility of conducting a longitudinal study in a population of middle-aged and elderly women. Future studies should aim to explore the clinical mechanisms underlying the interplay between depression and cognition in order to propose effective clinical interventions. Our findings have important implications for middle-aged and elderly women’s health research, suggesting that there is a bidirectional association between depression and cognition, with depression leading to worse cognitive status over time and prior cognitive impairment leading to subsequent depressive symptoms, which is an original finding for middle-aged and elderly women’s research. Based on our findings, it is recommended that society consider cognitive factors when treating depression in middle-aged and older women, and improve depressive states by improving educational and cognitive levels, as well as considering depressive states when treating cognitive decline.

## 5 Limitations

This study has several limitations. Firstly, all findings are based on self-reported data, which may deviate from objective reality. Self-rated memory or mood assessments could be subject to recall bias (e.g., participants may exaggerate or forget symptoms) and social desirability bias (e.g., concealing true feelings). Secondly, survey results might be influenced by the training level of investigators and environmental conditions. Variations in data collectors’ expertise, testing locations, and timing could affect response consistency and participants’ cognitive performance or emotional states. Thirdly, this study only examined the relationship between depression and cognition without adjusting for other confounding factors (e.g., age, education, and chronic diseases). Furthermore, the cognitive assessment tools in CHARLS have not undergone extensive, population-wide, and psychometrically robust validation, which may compromise the reliability of the findings and hinder comparability with prior studies. Fourthly, no adjustments were made for censored data. Subsequent studies should conduct sensitivity analyses to explore how different data handling approaches and subgroups affect variable relationships. Finally, the majority of participants in this study were married, resided in rural areas, and had only primary school education. Caution is warranted when generalizing these findings to broader populations.

## 6 Conclusions

Our study provides significant evidence on the relationship between depression and cognition in Chinese middle-aged and older women. The strength of our findings suggests that cognition and depression have a bidirectional relationship. The health of middle-aged and older women should be improved by treating depressive states and increasing cognition, which also suggests the value of implementing such clinical interventions.

## Data Availability

The datasets presented in this study can be found in online repositories. The names of the repository/repositories and accession number(s) can be found below: https://charls.charlsdata.com/pages/data/111/zh-cn.html.
